# Anti-endotoxic activity and structural basis for human MD-2·TLR4 antagonism of tetraacylated lipid A mimetics based on βGlcN(1↔1)αGlcN scaffold

**DOI:** 10.1177/1753425914550426

**Published:** 2015-07

**Authors:** Jose Antonio Garate, Johannes Stöckl, María del Carmen Fernández-Alonso, Daniel Artner, Mira Haegman, Chris Oostenbrink, Jesús Jiménez-Barbero, Rudi Beyaert, Holger Heine, Paul Kosma, Alla Zamyatina

**Affiliations:** 1 Institute of Molecular Modelling and Simulation, University of Natural Resources and Life Sciences, Vienna, Austria; 2 Institute of Immunology, Medical University of Vienna, Vienna, Austria; 3 Chemical and Physical Biology, CIB-CSIC, Madrid, Spain; 4 Department of Chemistry, University of Natural Resources and Life Sciences, Vienna, Austria; 5 Department for Biomedical Molecular Biology, Unit of Molecular Signal Transduction in Inflammation, Ghent University, Inflammation Research Center, VIB, Ghent, Belgium; 6 Research Group Innate Immunity, Research Center Borstel, Leibniz-Center for Medicine and Biosciences, Airway Research Center North (ARCN), Member of the German Center for Lung Research (DZL), Borstel, Germany

**Keywords:** Antagonist, glycolipids, lipid A, lipopolysaccharide, MD-2, molecular dynamics simulation, NMR, Toll-like receptor 4

## Abstract

Interfering with LPS binding by the co-receptor protein myeloid differentiation factor 2 (MD-2) represents a useful approach for down-regulation of MD-2·TLR4-mediated innate immune signaling, which is implicated in the pathogenesis of a variety of human diseases, including sepsis syndrome. The antagonistic activity of a series of novel synthetic tetraacylated bis-phosphorylated glycolipids based on the βGlcN(1↔1)αGlcN scaffold was assessed in human monocytic macrophage-like cell line THP-1, dendritic cells and human epithelial cells. Two compounds were shown to inhibit efficiently the LPS-induced inflammatory signaling by down-regulation of the expression of TNF-α, IL-6, IL-8, IL-10 and IL-12 to background levels. The binding of the tetraacylated by (*R*)-3-hydroxy-fatty acids (2 × C_12,_ 2 × C_14_), 4,4′-bisphosphorylated βGlcN(1↔1)αGlcN-based lipid A mimetic DA193 to human MD-2 was calculated to be 20-fold stronger than that of *Escherichia coli* lipid A. Potent antagonistic activity was related to a specific molecular shape induced by the β,α(1↔1)-diglucosamine backbone. ‘Co-planar’ relative arrangement of the GlcN rings was inflicted by the double *exo*-anomeric conformation around both glycosidic torsions in the rigid β,α(1↔1) linkage, which was ascertained using NOESY NMR experiments and confirmed by molecular dynamics simulation. In contrast to the native lipid A ligands, the binding affinity of βGlcN(1↔1)αGlcN-based lipid A mimetics to human MD-2 was independent on the orientation of the diglucosamine backbone of the synthetic antagonist within the binding pocket of hMD-2 (rotation by 180°) allowing for two equally efficient binding modes as shown by molecular dynamics simulation.

## Introduction

Activation of the TLR4-myeloid differentiation 2 (MD-2) complex, an essential component of the mammalian innate immune system, by LPS results in a systemic inflammatory response. One of the major concerns in TLR4 signaling is the ability of the active LPS–MD-2·TLR4 complex to overstimulate the innate immune system causing life-threatening health conditions such as sepsis syndrome and septic shock.^1,2^ Down-regulation of TLR4 signaling was shown to be beneficial for treatment of many chronic and acute inflammatory diseases such as asthma,^3^ arthritis,^4^ influenza^5^ and cancer.^6^ The ‘endotoxic principle’ of LPS resides in a glycophospholipid lipid A, which can be recognized and bound by the MD-2·TLR4 complex.^7^ The chemical structure of lipid A is based on the β(1→6)-linked 1-,4′-bisphosphorylated diglucosamine backbone, which is multiply acylated by (*R*)-3-hydroxy- or (*R*)-3-acyloxyacyl fatty acids. The immunobiological activity of lipid A depends on the number of factors, such as acylation pattern and the length of the lipid chains, as well as the presence or absence of the phosphate groups.^8^

Conformationally confined tetraacylated lipid A mimetics (LAM) based on the βGlcN(1↔1)αGlcN scaffold were designed and synthesized to investigate the molecular basis of the disruption of MD-2·TLR4-mediated inflammatory signaling.^9^ The idea of developing conformationally restricted lipid A mimetics was driven by the pioneering studies of the group of Seydel and Brandenburg, that disclosed a correlation between the endotoxic activity and the shape and volume of the hydrophobic region of lipid A enclosed in the lipid matrix, which was dependent on the inclination of the backbone with respect to the acyl chains.^10–13^ Furthermore, outstanding synthetic research by the groups of Fukase and Kusumoto also suggested the existence of a relationship between the molecular shape of a single lipid A molecule and its biological activity.^14–16^ In the inspiring seminal work of Zähringer and Grzesiek, the three-dimensional (3D) conformation of a monomeric LPS molecule was deciphered by intricate NMR experiments revealing a correlation between the relative orientation of the GlcN units of the βGlcN(1→6)GlcN lipid A backbone in differently acylated LPS structures.^17^

**Figure 1.**
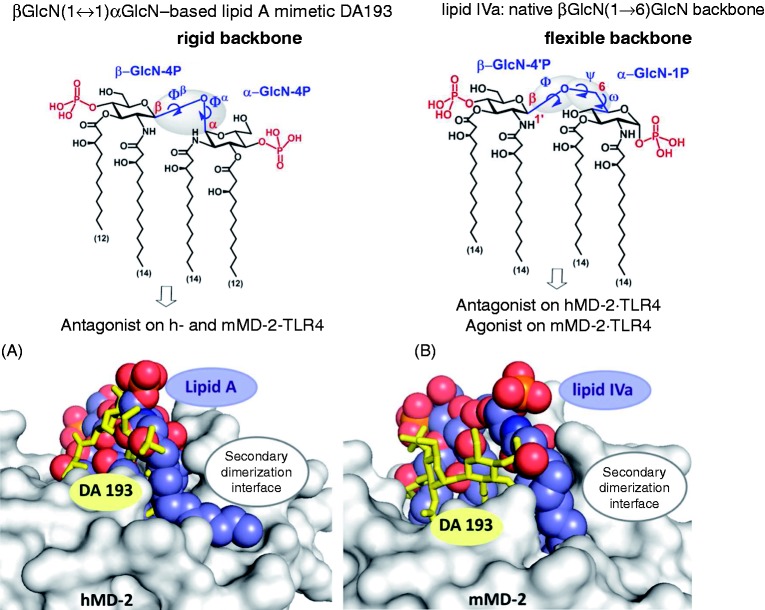
(Top) Chemical structure of βGlcN(1↔1)αGlcN-based LAM DA193 having a rigid two-bond linked β,α(1↔1) diglucosamine backbone in comparison with the flexible three-bond linked β(1→6) diglucosamine backbone of native lipid A and lipid IVa. (Bottom) Antagonist DA193 (stick model, yellow) in the binding pocket of h- and mMD-2. (A) Superimposition of DA193^hMD-2^ (obtained by molecular dynamic simulations) and *E. coli* lipid A^hMD-2·TLR4^ (space-filling model) in the binding pocket of hMD-2 (PDB code: 3FXI). All four acyl chains of DA193 are fully buried in the interior of the binding pocket of MD-2, whereas the 2 *N*-acyl chain of lipid A is exposed on the surface of the protein at the secondary dimerization interface. (B) Superimposition of DA193^mMD-2^ (obtained by molecular dynamic simulations) and agonist lipid IVa^mMD-2·TLR4^ (space-filling model) in the binding pocket of mMD-2 (PDB code: 3VQ1). DA193 submerges much deeper into the binding pocket of mMD-2 compared to the agonist lipid IVa. Images were generated with PyMol.

In the βGlcN(1↔1)αGlcN LAMs the inherently flexible three-bond β(1→6) glycosidic linkage of the carbohydrate backbone of native lipid A is displaced by a rigid two bond βα(1↔1) glycosidic linkage (Figure 1). Thus, βGlcN(1↔1)αGlcN-based LAMs represent conformationally restricted counterparts of the biosynthetic precursor of *Escherichia coli* lipid A, lipid IVa, which displays species-specific activity acting as antagonist on human (h) MD-2·TLR4 and as agonist on mouse (m) MD-2·TLR4 complex.^18^ We have recently demonstrated that, in contrast to lipid IVa, the βGlcN(1↔1)αGlcN LAMs exhibit dose-dependent antagonistic activity on both h- and mMD-2·TLR4 complexes (Figure 1).^9^ Matching results were obtained in HEK293 cells transiently transfected with membrane CD14 (mCD14)/hMD-2·TLR4 (HEK-Blue, detection by measuring of induction of secreted embryonic alkaline phosphatase) and in HEK293 cells transfected with hMD-2·TLR4 only (NF-κB luciferase reporter assay), thus confirming the MD-2 specificity of the action of synthetic antagonists.

To further investigate the antagonistic properties of tetraacylated βGlcN(1↔1)αGlcN-based lipid A mimetics in human cells, we examined the ability of six variably acylated βGlcN(1↔1)αGlcN LAMs to inhibit LPS-induced pro-inflammatory responses in human macrophage-like cell line (THP-1), in human epithelial cells and in human immune cells [dendritic cells (DCs)].

**Figure 2.**
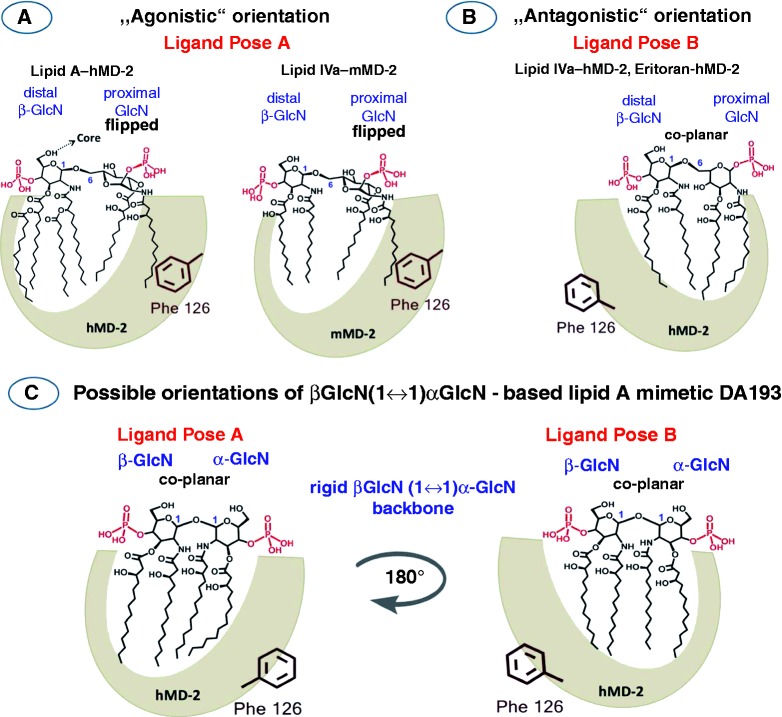
Orientations of the lipid A ligands within the binding pocket of MD-2. (A) Schematic representation of the orientation of the agonistic ligands resolved in the co-crystal structures: *E. coli* lipid A^hMD-2·TLR4^ (PDB code: 3FXI) and lipid IVa^mMD-2·TLR4^ (PDB code: 3VQ1) wherein the proximal (reducing) GlcN ring of the βGlcN(1↔6)GlcN backbone faces the Phe126 loop. Phe126 is shifted inward to stabilize the exposure of the 2*N*-acyl chain on the surface of MD-2 and to allow the dimerization with the second MD-2*·TLR4* complex. (B) Orientation of the antagonistic ligand lipid IVa ^hMD-2^ (PDB code: 2E59) wherein the distal (non-reducing) GlcN ring faces the Phe126 loop. Phe126 is oriented outwards and exposed to solvent, which prevents the dimerization of two receptor complexes. (C) Two modeled orientations of DA193.

One of the most striking and still not clarified aspects disclosed in the co-crystal structures of MD-2·TLR4 ligand complexes is that the orientation of the agonistic ligands (such as hexaacylated *E. coli Ra*-LPS^hMD-2·TLR4^, *E. coli Re*-LPS^mMD-2·TLR4^ and tetraacylated lipid IVa^mMD-2·TLR4^)^18,19^ is inverted by 180° compared with the positioning of the MD-2-bound antagonistic ligands (Eritoran^hMD-2·TLR4^ and lipid IVa^hMD-2^)^20,21^ (Figure 2A, B).^22^ It has been shown that the exposure of a single acyl chain of an agonist on the surface of MD-2 drives the activation of MD-2·TLR4 complex by supporting its homo-dimerization.^18,23^ We have previously proposed that not only the chemical structure of lipid A, but also the 3D molecular shape of MD-2-bound lipid A (or lipid A-like ligands) determines the immuno-biological activity on TLR4.^9^ Conformational rigidity of βGlcN(1↔1)αGlcN scaffold wherein two GlcN rings are co-planar oriented precludes the “flipping” of the proximal GlcN moiety (as in the native MD-2-bound lipid A) and, therefore, the exposure of the long-chain acyl residue on the surface of the protein. Based on the fact that βGlcN(1↔1)αGlcN LAMs are excellent antagonists on both h- and mMD-2·TLR4 complexes, we supposed that, in contrast to native lipid A ligands, the binding affinity and the MD-2-specific activity of the βGlcN(1↔1)αGlcN LAMs should not relate to the geometric orientation of their (1↔1)-linked diglucosamine backbone within the binding pocket of MD-2 (rotation by 180℃) (Figure 2C). To support this hypothesis, we performed molecular dynamics simulation of βGlcN(1↔1)αGlcN LAM DA193 (Figure 1), the most potent hTLR4 antagonist in the series, in two possible orientations within the binding pocket of hMD-2 (pose A wherein the α−GlcN ring faces the Phe126 loop and pose B wherein the β-GlcN ring faces the Phe126 loop of hMD-2 as in Figure 2C). To draw definite conclusions about the 3D molecular shape of the non-reducing βGlcN(1↔1)αGlcN backbone, the conformation of variably acylated LAMs in solution was studied by NMR. The NMR NOESY data describing the spatial arrangement of two GlcN rings in the conformationally confined β,α−(1↔1)-linked scaffold were correlated to the geometric characteristics of the MD-2-bound lipid A mimetic DA193 obtained by molecular dynamics simulation.

## Materials and methods

### Biological assays

#### Reagents

THP-1 cells were a kind gift of R. De Vos (Roche Ghent); the cell culture medium RPMI 1640 (Life Technologies, Carlsbad, CA, USA) was supplemented with 2 mM L-glutamine, 100 U/ml penicillin, 100 µg/ml streptomycin and 10% fetal calf serum (Sigma-Aldrich, St. Louis, MO, USA). Recombinant human granulocyte/macrophage colony-stimulating factor (GM-CSF) and IL-4 were kindly provided by Jens Gerwien (Novo Nordisk A/S, Malov, Denmark). LPS from *E. coli* (serotype O127:B8 and serotype O111:B4) were purchased from Sigma-Aldrich. The following murine mAbs were generated in the laboratory of JS (Institute of Immunology, Medical University of Vienna): negative control mAb VIAP (calf intestinal alkaline phosphatase-specific); DF272 (B7-H1); 1/47 [major histocompatibility complex (MHC) class II], VIT6b (CD1a); 7-236 (CD169); and VIM13 (CD14). Hybridomas producing mAb W6/32 (MHC class I) and were obtained from the ATCC (Manassas, VA, USA). The CD14 mAb (MEM18) was kindly provided by An der Grub (Bio Research GmbH; Kaumberg, Austria), and the CD19 mAb (HD37) was a gift from G. Moldenhauer (Department of Molecular Immunology, DFKZ Heidelberg). MAb BU63 (CD86) were purchased from Invitrogen (Carlsbad, CA, USA). βGlcN(1↔1)αGlcN LAMs were solubilized as follows: a 1-mg/ml solution in DMSO was prepared first; aliquots of this stock solution were diluted with cell medium supplemented by 10% FCS to obtain aqueous solutions where the amount of DMSO did not exceed 0.01% and 0.1% at a concentration of antagonist 100 and 1000  ng/ml, respectively.

### Assay in THP-1 cells

THP-1 cells [human acute monocytic leukemia cell line induced for monocytic differentiation with 12-*O*-tetradecanoylphorbol-13-acetate (TPA)] were seeded in 96-well plates at 100,000 cells/well in 150 µl and simultaneously stimulated with 200 nM TPA for 24 h.^24^ On the next day the cells were washed twice with DMEM and with serum to discard the cells that did not adhere, refreshed with 200 µl fresh medium and left for 1 h to recover. Cells were stimulated with 100 ng/ml *E. coli* O111:B4 LPS, which was added as a solution in 10 µl (DMEM + 10% FCS). βGlcN(1↔1)αGlcN LAMs were dissolved in DMSO (1 mg/ml) and further diluted with DMEM + 10% FCS to reach the concentrations of 100 ng/ml and 1000 ng/ml. Corresponding doses of synthetic antagonists were added to the cells immediately after stimulation with LPS. The total volume of the well after stimulation/inhibition reached 220  µl. The cells were incubated for 18 h and the supernatants were analyzed for TNF-α by ELISA. At the end of the incubation period, cells were scored microscopically for potential effects of the βGlcN(1↔1)αGlcN-LAMs on cell growth or survival. None of the compounds was found to be toxic at the concentrations and incubation times used.

### Assay in DCs

#### Cell preparation and stimulation

PBMCs were isolated from heparinized whole blood (buffy coats) of healthy donors purchased from the Red Cross in Austria by standard density gradient centrifugation with Ficoll-Paque (Pharmacia Biotech, Piscataway, NJ, USA). Subsequently, monocytes and T cells were separated by magnetic sorting using the MACS technique (Miltenyi Biotec, Cologne, Germany) as previously described.^25^ Monocytes were enriched using the biotinylated CD14 mAbs VIM13 and MEM18 (purity >95%). Purified T cells were obtained through negative depletion of CD11b, CD14, CD16, CD19, CD33 and MHC class II-positive cells with the respective mAbs. DCs were generated from CD14^+^ monocytes cultured in the presence of GM-CSF (50 ng/ml) and IL-4 (100 U/ml) for 6 d. Maturation of DCs was induced by adding 10 ng/ml *E. coli* O127:B8 LPS for 24 h in the presence or absence of βGlcN(1↔1)αGlcN-LAMs. Thus, human PBMCs were cultured for 6 d in GM-CSF and IL-4 to receive immature monocyte-derived DCs and were then stimulated with 10 ng/ml LPS with or without treatment with synthetic antagonists which were used at a concentration of 100, 500 and 1000 ng/ml. After 24 h cells were harvested and the surface expression level of the indicated markers (Supplementary Figure S1) was measured by flow cytometry.

#### Immunofluorescence analysis

For membrane staining, cells (5 × 10^5^) were incubated for 30 min at 4℃ with unlabeled mAbs at a concentration of 20  µg/ml. Staining of DCs was performed in the presence of human IgG Abs (20 mg/ml; Beriglobin; Aventis Behring Marburg, Germany). After washing cells twice with ice-cold PBS containing 1% BSA, binding of the primary mAb was visualized by the use of Oregon Green-conjugated goat anti-mouse Ab from Molecular Probes (Eugene, OR, USA). Cells were then washed three times with PBS/BSA. Membrane fluorescence was analyzed on a FACSCalibur flow cytometer (BD Biosciences, San Jose, CA, USA) supported by CellQuest-Pro software (BD Biosciences). The exclusion of dead cells was performed by the addition of propidium iodide.

#### Determination of cytokine production

DCs were treated as indicated, and after 24 h the supernatants were harvested and analyzed by Luminex (Austin, TX, USA) testing for the presence of TNF-α, IL-10, IL-12p70 and IL-6 using specific matched-pair Abs and recombinant cytokines as standards (eBioscience, San Diego, CA, USA) as described.^26^ Cytokine measurements were performed in duplicates using the Luminex System 100. Results are representative of three independent experiments (mean values of triplicate examinations ± SD are presented in Figures 4 and 5).

### Assay in epithelial cells

Beas-2 b (an Ad12SV40-transformed human bronchioepithelial cell line) or Calu-3 cells (a human lung epithelial cell line; both from the ATCC) were seeded in 96-well plates at 100,000 cells/well in 100 μl of complete medium [RPMI1640 (PAA Laboratories, Pasching, Austria), 1% PS (PAA Laboratories, Pasching, Austria), 10% FCS (Biochrom, Berlin, Germany)]. On the next day, the cells were washed once with complete medium. Before stimulation with 10 ng/ml *E. coli* O111:B4 LPS, cells were pretreated with 100 or 1000 ng/ml βGlcN(1↔1)αGlcN LAMs for 1 h. Synthetic antagonists were dissolved in DMSO (100 µg/ml) and further diluted with complete medium to reach the concentrations of 100 ng/ml and 1000 ng/ml. The total volume of the well after stimulation/inhibition reached 200 μl. The cells were incubated for 20–24 h and the supernatants were analyzed for IL-8 and IL-6 by ELISA (Life Technologies).

### NMR spectroscopy

Two sets of experiments were performed using DMSO-d_6_ or D_2_O/deuterated SDS micelles as solvent. NMR experiments were recorded at 298 K on a Bruker AV500 spectrometer (Bruker, Billerica, MA, USA). Spectra were obtained with standard sequences from the TOPSPIN software package (Bruker). For the DMSO experiments, a ∼ 2 mM concentration for the βGlcN(1↔1)αGlcN-LAMs was employed. For experiments in the presence of deuterated SDS micelles, 23.80 μl of a stock solution of DA257 (4.2  mM in DMSO-d_6_) was mixed with 45.5 μl of SDS and further diluted with D_2_O to give a final concentration of 0.5 ml of the ligand in the presence of SDS (20 mM). The components were mixed up by vortexing. The Bruker pulse sequence *noesygpph19* was used for the NOESY experiments. Mixing times of 250 and 350 ms were used. The two-dimensional spectra were acquired with 1 K–2  K data points in the F2 dimension and 256 data points in the F1 dimension. The residual water signal was suppressed by presaturation. Prior to Fourier transformation, all spectra were multiplied with a sine-squared function.

### Molecular dynamics simulation

Model building is described in the Supporting Information.

#### Simulation setup

All simulations were performed with the program NAMDv2.9.^27^ The Particle Mesh Ewald method was used for long-range electrostatics within a relative tolerance of 1^.^10^−6^.^28^ A cut-off distance of 1.2 nm was applied to real-space Ewald interactions and for the van der Waals interactions, with a smooth switching function applied between 1.0 and 1.2 nm. Multiple time steps were used with time steps of 2 fs for bonded interactions, 2 fs for short-range non-bonded interactions and 4 fs for the full electrostatics evaluation using the r-RESPA method. All production runs were performed at constant temperature and pressure, with reference values of 298 K and 1 atm, using the Nose-Hoover and Langevin piston methods with damping coefficients of 1 ps^–1^.^29,30^ The SHAKE algorithm was applied to constrain bond lengths to all hydrogen atoms.^31^ After an initial thermalization, all simulations were run for 11 ns, discarding the first ns.

#### Analysis of molecular dynamics simulations

Hydrogen bonds of the ligands with the solvent and the protein were identified by geometric criteria: a donor–acceptor pair is considered to be hydrogen bonded if the donor–acceptor distance is < 0.35 nm and the donor–hydrogen–acceptor angle is >150°. Salt bridges between the negatively charged phosphates of the lipids and the positively charged residues of MD-2 were determined using a cut-off of 0.7  nm between the central phosphorous atoms and the C_z_ of arginine or the N_z_ of lysine, respectively. Free energy difference (ΔG_bind_) calculations were computed using the linear interaction energy (LIE) method,^32^ with the empirical parameters α = 0.18 and β = 0.09.^33^ As NOE intensities for slowly tumbling molecules are proportional to the inverse distance to the third power, average distances from the simulations were calculated as <r^−3^>^−1/3^, with angular brackets indicating an ensemble average.

## Results

### Antiendotoxic potential of βGlcN(1↔1)αGlcN-based lipid A mimetics in human macrophage-like cell line THP-1

**Figure 3.**
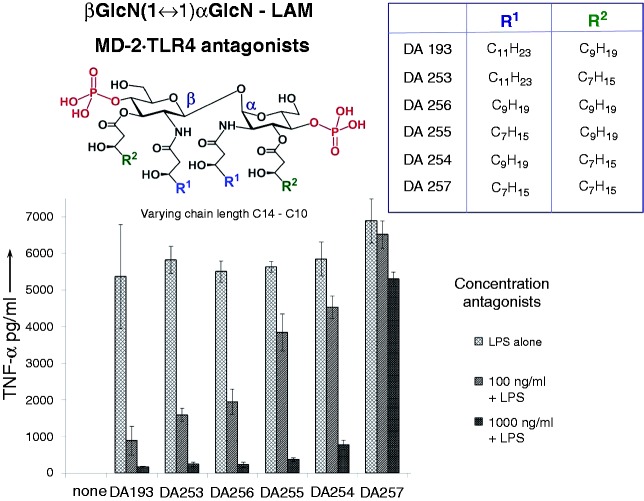
Inhibition of the expression of TNF-α by βGlcN(1↔1)αGlcN-LAMs in THP-1 cells stimulated with 100 ng/ml *E. coli* O111:B4 LPS.

Six variably acylated βGlcN(1↔1)αGlcN-based lipid A mimetics were examined for the inhibition of expression of TNF-α in the human monocytic cell line THP-1 that was differentiated into macrophages by TPA treatment (Figure 3). Though THP-1 cells are derived from the blood sample of a patient with acute monocytic leukemia, this cell line resembles primary monocytes and macrophages from healthy donors.^24^

Macrophage-like THP-1 cells express MD-2, mCD14 (which is required for the transfer of monomeric LPS to the MD-2·TLR4 complex) and a variety of cell surface receptors, including TLR4.^34^ The cells were stimulated by *E. coli* LPS and subsequently (10 min) treated with variable concentrations of synthetic antagonists. The LPS-induced production of TNF-α was suppressed to the background levels by all βGlcN(1↔1)αGlcN LAMs at a concentration of 1 µg/ml except for the short-chain DA257 (4 × C_10_ acylated), whereas DA193 (2 × C_14_, 2 × C_12_ acylated) was the most potent antagonist providing 90% inhibition at a concentration of 100 ng/ml.

### Anti-endotoxic activity of βGlcN(1↔1)αGlcN-based lipid A mimetics in human DCs

DCs, which are professional pathogen-decoding APCs, reside in peripheral tissues as immature APCs that can be activated by PAMPs,^35^ and, in particular, by LPS.^36^ LPS-stimulated DCs mature to the competent T cells by translocating MHC–peptide complexes to the cell surface and up-regulating co-stimulatory cell surface receptors. Besides, activated DCs produce a number of cytokines engaged in elimination of infection and modulation of the T-cell responses.^37^

**Figure 4.**
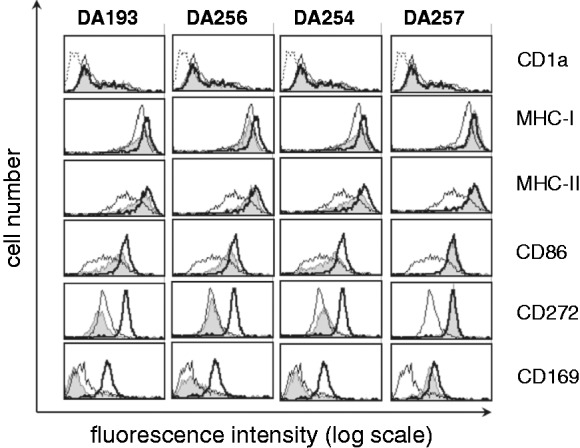
Inhibition of expression of maturation markers induced by *E. coli* LPS in monocyte-derived DCs by βGlcN(1↔1)αGlcN-based LAMs. Immature monocyte-derived DCs (open histograms, thin line) were stimulated in parallel for 24 h with 10 ng/ml LPS either with (open histograms, thick line gray histograms) or without pretreatment with synthetic antagonists (gray histograms). βGlcN(1↔1)αGlcN LAMs were used at a concentration of 500 ng/ml. The dotted line in the CD1a histogram represents VIAP staining (isotype control). Results are representative of three independent experiments.

The ability of selected βGlcN(1↔1)αGlcN LAMs, which revealed the highest potency in inhibiting the activation of NF-κB in the HEK-blue cells,^9^ to interfere with LPS-induced NF-κB and IFN regulatory factor (IRF) inflammatory pathways by inhibiting the up-regulation of specific surface markers on DCs was initially assessed. To test the impact of βGlcN(1↔1)αGlcN LAMs on DC maturation, immature, monocyte-derived DCs were treated with LPS with or without the addition of four variably acylated βGlcN(1↔1)αGlcN LAMs. DCs treated with LPS acquired a characteristic morphologic phenotype and displayed specific markers of mature DCs when analyzed by flow cytometry (Figure 4). Treatment of DCs stimulated by *E. coli* LPS with the synthetic antagonists DA193, DA254 and DA256 did not alter the expression of characteristic cell surface markers on DCs such as CD1a, but blocked the up-regulation of the surface markers induced by LPS, including co-stimulatory molecules CD86, as well as the Ag presenting structures, such as MHC class I and MHC class II, which are necessary for the induction of an adaptive immune response, to the background levels. Furthermore, LPS-induced up-regulation of the inhibitory B7-H1 (CD272) and Sialoadhesin (CD169) were also completely inhibited by DA193, DA254 and DA256. As CD169 is known to be indirectly upregulated in DCs due to release of type-I IFNs,^38^ our data also demonstrate that DA193, DA254 and DA256 are potent inhibitors not only of the NF-kB pathway, but also of the IRF pathway induced by LPS-triggered TLR4 stimulation. In contrast, treatment of DCs with the short-chain lipid A mimetic DA257, which was shown to be inactive in inhibiting LPS signaling in THP-1 cells, did not alter expression of the typical differentiation marker CD1a,^25,35^ and failed to prevent the induction of LPS-induced DC maturation. None of the applied synthetic compounds exerted cytotoxic effects on DCs (as determined by propidium iodide staining) or induced morphological changes or cell death either alone or in combination with LPS at the concentrations used.

**Figure 5.**
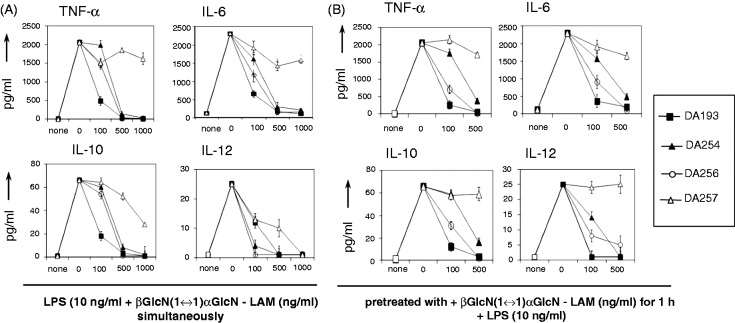
Inhibition of expression of cytokines by βGlcN(1↔1)αGlcN-based LAMs in monocyte-derived DCs induced by *E. coli* O127:B8 LPS. (A) βGlcN(1↔1)αGlcN-LAMs (at the concentrations of 100, 500 and 1000 ng/ml) were added simultaneously with LPS (10 ng/ml). (B) Cell culture was pre-incubated with βGlcN(1↔1)αGlcN LAMs for 1 h (at concentrations of 100, 500 and 1000 ng/ml) prior to addition of LPS. Mean values of triplicate examinations ± SD are presented.

The capacity of the synthetic TLR4 antagonists to impede the LPS-induced expression of pro-inflammatory cytokines in human DCs was assessed by incubation of immature monocytes-derived DCs with LPS in the presence of βGlcN(1↔1)αGlcN LAMs. The βGlcN(1↔1)αGlcN LAMs DA193, DA254, DA256 and DA257 were examined for efficacy in prohibiting the induction of cytokines that are normally released by DCs during acute infections or upon *in vitro* treatment with LPS. The expression of pro-inflammatory cytokines TNF-α, IL-6, the immunosuppressive factor IL-10 and the important cytokine for effector T-cell responses, IL-12, were completely suppressed by application of DA193 at a concentration of 100 ng/ml, whereas DA256 and DA254 were somewhat less efficient, exerting similar inhibitory activity at a concentration of 500 ng/ml (Figure 5). The anti-endotoxic effects were observed independently of the sequence of addition of antagonists and LPS to the cell culture [simultaneous addition of synthetic antagonists and LPS to the cell culture (Figure 5A) or pretreatment with synthetic antagonist for 1 h prior to stimulation with LPS as in Figure 5B]. Short-chain DA257 was virtually inactive, whereas it was able to reduce only the release of IL-12 caused by LPS.

### Evaluation of antagonistic activity of βGlcN(1↔1)αGlcN-based lipid A mimetics in human epithelial cells

**Figure 6.**
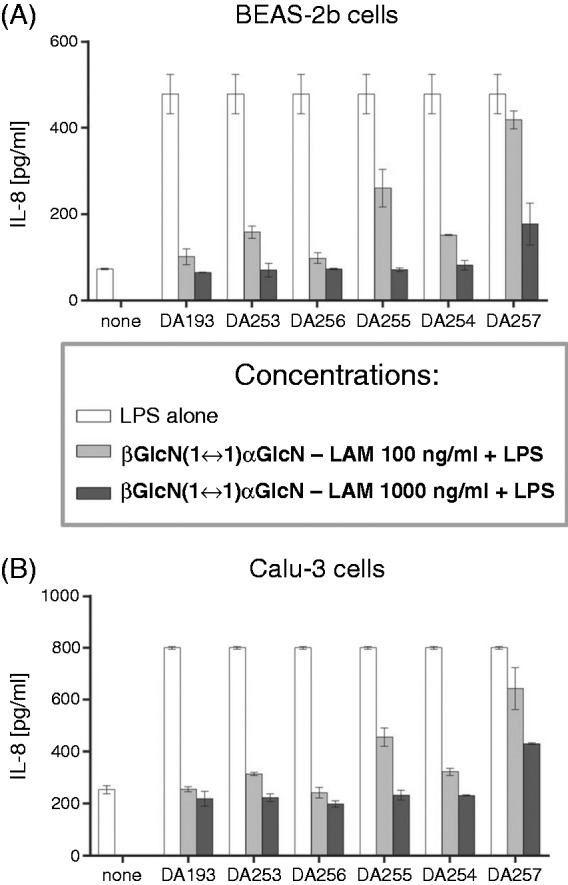
Inhibition of production of IL-8 by βGlcN(1↔1)αGlcN LAMs in human epithelial cell lines (A) BEAS-2b and (B) Calu-3 stimulated by addition of 10 ng/ml *E. coli* O111:B4 LPS.

We assessed the antagonistic activity of the βGlcN(1↔1)αGlcN LAMs in two different human lung epithelial cell lines, BEAS-2B and Calu-3, which express TLR4 and MD-2, but do not express mCD14. With respect to the inhibition of IL-8 release, both cell lines behaved nearly identically (Figure 6A, B) revealing that DA193 and DA256 were the most potent antagonists, with a concentration of 100 ng/ml sufficient to reduce cytokine release to almost baseline levels (> 90% inhibition). DA253 and DA254 were somewhat less effective, providing > 80% inhibition at 100 ng/ml antagonist. At the same concentration, shorter-chain DA255 reduced cytokine release only by 50–60%. However, all compounds except for DA257 completely suppressed activation when used at a concentration of 1000 ng/ml. A nearly identical pattern of the antagonistic capacity could be seen for the inhibition of IL-6 release (data not shown).

### Assessment of the conformation about β,α(1↔1) glycosidic linkage by NMR

**Figure 7.**
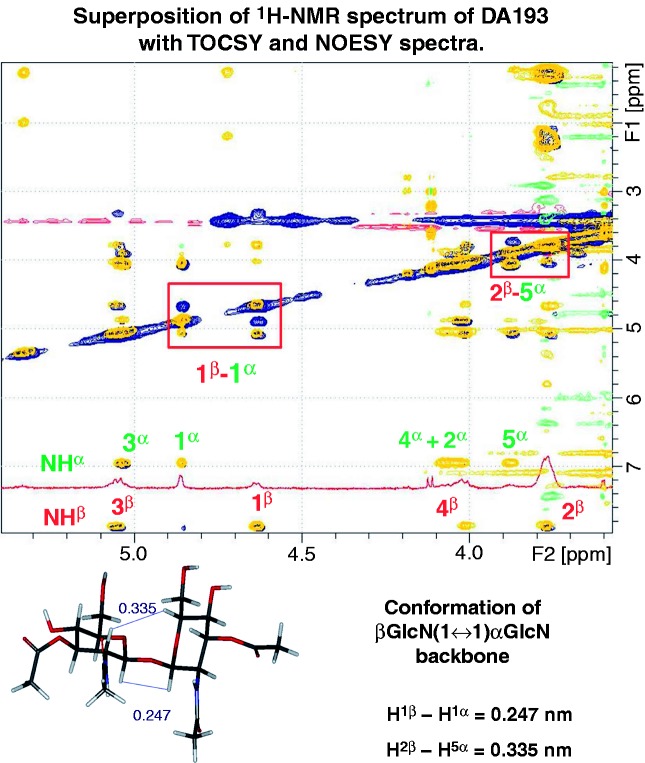
Superposition of ^1^H-NMR spectrum of DA193 in DMSO-d_6_ (red) with TOCSY (yellow) and NOESY (blue) spectra. The computed proton–proton distances for the diglucosamine scaffold of DA193 are shown.

The potent antagonistic activity of the βGlcN(1↔1)αGlcN LAMs was attributed to a specific molecular shape of the conformationally confined βGlcN(1↔1)αGlcN backbone. Therefore, the experimental assessment of the conformation about βα−(1↔1) glycosidic linkage by NOESY experiments of variably acylated synthetic glycolipids (DA193, DA256 and DA257) was performed.^39^ Given the poor solubility of glycolipids in water, the NMR data were acquired first in DMSO-d_6_ followed by D_2_O–SDS micelles, taking advantage of the fact that the basic conformational features of glycolipids in different solvent media are preserved.^40,41^ The analysis of the vicinal coupling constants indicated that both d-GlcN pyranose rings adopted the expected chair conformation,^41,42^ independently of the length of the 2 -*N*- and 3-*O*-acyloxy chains. Moreover, the chemical shifts of the sugar protons were basically identical in all three compounds (online Supplementary Figure S1).^9^ The cross peaks in the NOESY experiments were already in the negative NOE regime, indicating that the molecules display slow tumbling in DMSO solution. The analysis of the NOEs indicated that a *syn*Φα/*syn*Φβ geometry was present in the solution, given the large intensity of the H^1^α/H^1^β cross peaks for all three disaccharides (Figure 7; online Supplementary Figure S2). Such NOE is conclusive for a double *exo*-anomeric conformation around both glycosidic torsions.^43^ A full relaxation matrix analysis of the NOE intensities suggested that the corresponding H^1^α–H^1^β distances lay between 0.23 and 0.25 nm, which was similar for both long-chain DA193 and the shorter-chain DA256 and DA257 compounds (online Supplementary Figure S3). A peak H^5^α/H^2^β NOE could also be detected corresponding to an experimental distance H^5^α–H^2^β of about 0.35 nm.

As DMSO is known to disrupt hydrophobic interactions between lipid chains that could influence the overall conformation of the molecule, we analyzed the conformational behavior of lipid A mimetics in D_2_O supplemented by SDS micelles. Given the similarity of the NMR spectra of variably acylated βGlcN(1↔1)αGlcN LAMs in DMSO, the analysis was carried out for the shorter-chain DA257, taking advantage of its better solubility in water/SDS. Notably, the resolution of the ^1^H-NMR spectrum of DA257 in the SDS micelle environment was significantly improved with respect to that in DMSO (online Supplementary Figure S4). Acquisition of the NOESY spectrum allowed for assignment of the strong cross peak H^1α^/H^1β^ and assessment of the conformation of glycosidic linkage, which was similar to the value obtained in DMSO. The cross peaks were already negative, indicating the large size of the aggregates in solution and, again, the predominant *syn*Φα/*syn*Φβ conformation of glycosidic linkage was confirmed, which was in agreement with previous conformational studies on trehalose-like molecules.^39,43^

### Molecular dynamics simulation of antagonist DA193 in the binding pocket of hMD-2

Molecular dynamics simulations of DA193, the most potent hMD-2·TLR4 antagonist in the series of βGlcN(1↔1)αGlcN LAMs, were performed to gain a deeper insight into the molecular basis of the ligand recognition by hMD-2. Two possible orientations (rotation by 180°) of DA193 in the binding pocket of hMD-2 were simulated and compared in geometric characteristics and binding affinities to the modeled MD-2-bound native ligands *E. coli* lipid A^hMD-2·TLR4^ and antagonist lipid IVa^hMD-2^, also simulated in two orientations (the orientation reported in the co-crystal structures and the inverted one).^44^ The energetic penalties arising upon ligand binding by the protein and the non-covalent interactions occurring at the binding interface were also assessed.

**Figure 8.**
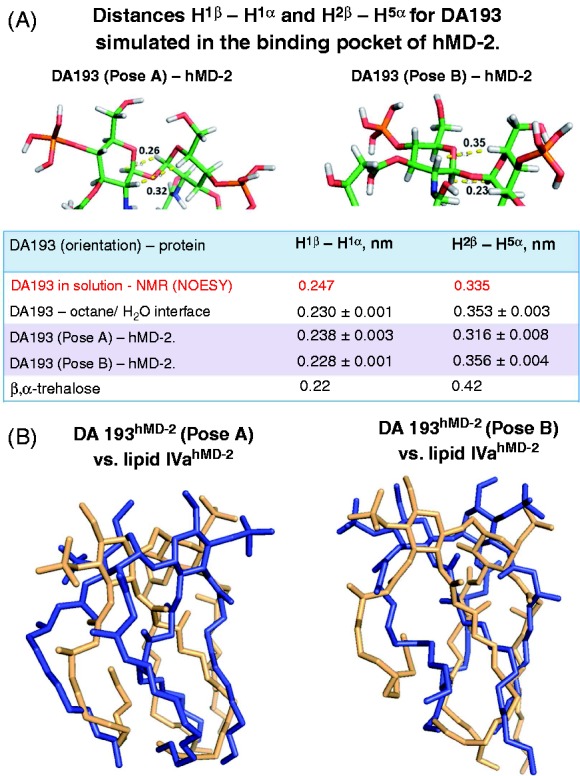
(A) Conformation of the βGlcN(1↔1)αGlcN backbone of DA193 simulated in the binding pocket of hMD-2 and at an octane–water interface compared with the conformation experimentally obtained from the NOESY NMR experiments of the ligands in solutions. Images were generated with PyMol. Averages of MD simulations were calculated as <r^-3^>^–1/3^ and error estimates were obtained from block averaging. NMR distances are considered as upper bounds such that the length longer than the experimental value is a violation, while any value that is lower than the experimental value is allowed. (B) Superimposition of antagonistic lipid IVa^hMD-2^ (light orange, PDB code: 2E59) with (A) simulated DA193^hMD-2^ (blue) in pose A and (B) with simulated DA193^hMD-2^ in pose B; hMD-2 is not shown for clarity.

The calculated average values of the Φα/Φβ torsions and, consequently, H^1^α–H^1^β and H^2^β–H^5^α distances for the protein-bound DA193, as well as for DA193, placed at the octane–water interface were in excellent agreement with the experimentally determined (NOESY) upper bounds, whereas the maximum violation amounted to 0.018 nm (Figure 8A). Overall, the calculated H^1^α–H^1^β and H^2^β–H^5^α distances and the corresponding torsions only insignificantly deviated from the experimentally observed values. Moreover, the 3D arrangement of the diglucosamine backbone of DA193 minimized in the protein-bound state only slightly deviated from the conformation of β,α-trehalose found in the x-ray structure,^45^ which can be explained by the rigidity of βα(1↔1)glycosidic linkage and the independence of the conformation on the nature and number of substituents (online Supplementary Figure S5).

The distance between 4 and 4′ phosphate groups in DA193 only marginally differed from the P1–P4′ distances in the native ligands found in the co-crystal structures (online Supplementary Table S1). Apparently, the plasticity of the phosphate groups on one side and the rigidity of β,α−(1↔1) glycosidic linkage which keeps two (1↔1)-connected GlcN rings in a nearly co-planar arrangement on the other side readily compensate for the shorter two-bond linkage between the GlcN rings in DA193 compared with the three-bond (1→6) glycosidic linkage in native lipid A and lipid IVa (Figure 8B).

**Figure 9.**
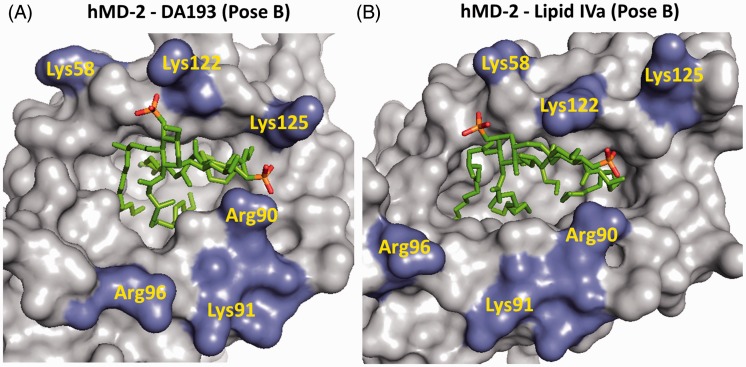
Electrostatic interactions at the rim of the binding pocket of hMD-2. (A) 4,4′-phosphate groups (in red) of modeled DA193 (Pose B) are involved in intense ionic contacts with Lys122, Lys125, Arg90 and Lys58; moderate interactions are detected with Arg96. (B) 1, 4′-Phosphate groups of lipid IVa (Pose B, corresponds to PDB code 2E59) establish strong ionic bridges with Lys122, Arg90 and Lys58. Images were generated with PyMol.

We have also compared the key interactions of the lipid A ligands with hMD-2 such as hydrogen bonding and ionic bridges. The average total number of hydrogen bonds was slightly higher for lipid A^hMD-2^ and lipid IVa^hMD-2^ than for DA193^hMD-2^, whereas the average loss of hydrogen bonds upon binding by hMD-2 amounted to 1.8 for lipid A, 0.5 for lipid IVa and 1.1 for DA193 (Table 1). Comparing the number of salt bridges that are formed between the phosphate groups of the ligands and positively charged Lys and Arg at the rim of the binding groove of MD-2 revealed a relatively low average count of electrostatic interactions for *E. coli* lipid A^hMD-2^ and lipid IVa^hMD-2^, in contrast to intensive ionic contacts provided by hMD-2-bound DA193 (Table 1; Figure 9). The differences in the binding free energy were considered by comparing the free energy of the protein-ligand bound states (Poses A and B) with the unbound ligands at an octane–water interface. The small negative values for ΔG_bind_ indicate that the ligand shows favorable interactions with the protein (Table 2). Accordingly, DA193 demonstrated the strongest affinity for hMD-2, followed by lipid IVa and lipid A. In terms of dissociation constants, DA193 is estimated to bind to hMD-2 20-fold more strongly than lipid A and three-fold more effectively than lipid IVa.

**Table 1.** Average occurrence of hydrogen bonds and salt bridges for lipid A, lipid IVa and DA193 in complex with hMD-2, together with the atom positional root mean square displacement (RMSD) of Phe126.^a^

MD-2 ligand	Pose (orientation)	H-bonds^b^ (nm)	Salt bridges (nm)	RMSD^c^ F126 (nm)
Lipid A	A as in 3FXI	12.4 ± 0.1	0.3	0.18
Lipid A	B	13.0 ± 0.3	0.5	0.37
Lipid IVa	A	13.3 ± 0.2	0.2	0.69
Lipid IVa	B as in 2E59	12.8 ± 0.1	0.6	0.38
DA193	A	11.4 ± 0.1	2.1	0.43
DA193	B	12.7 ± 0.2	1.0	0.31

a Error estimates are calculated from block averages.

b Average total number of hydrogen bonds, including the protein and the solvent. In pure water or octane–water environment, the number of hydrogen bonds is, on average, 14.5 (lipid A); 13.6 (lipid IVa); 12.8 (DA193) nm.

c Atom-positional RMSD of Phe126 at the end of the simulations with respect to the initial MD-2 structure calculated after a rotational fit on the Cα atoms of the MD-2 backbone. All simulations were started from the agonistic MD-2 structure (3FXI). The Phe126 remained relatively close to its original position (small RMSD) in the simulations of lipid A^hMD-2^ in pose A, while the crystallographically not-observed binding poses (lipid A in pose B and lipid IVa in pose A) induced a larger conformational change of Phe126. Larger deviations are observed when hMD-2 antagonist (lipid IVa or DA193) is placed in an agonistic protein structure (3FXI). Thus, the dynamic behavior of Phe126 was in line with the antagonistic role of DA193.

**Table 2.** Relative free energy differences in kcal/mol as calculated from molecular dynamics simulations.^a^

	Lipid A	Lipid IVa	DA193
ΔG_bind_^b^	−1.3 ± 0.4	−2.4 ± 0.4	−3.0 ± 0.4
ΔG_pose_ (A>B)^c^	1.6 ± 0.3	2.2 ± 0.4	−0.1 ± 0.4

a Error estimates are calculated from block averages.

b Free energy of binding as estimated by the LIE method: ΔG_bind_ = αΔ<V_vdw_> + βΔ<V_el_>, where <V_vdw_> and <V_el_> are the average ligand-surrounding van der Waals and electrostatic interaction energies, Δindicates the difference between the ligand-bound and unbound simulations; α = 0.18 and β = 0.09, using exponential averages of different poses.^48^ The free energy of binding is logarithmically dependent on the dissociation constant K_d_: ΔG*_bind__ _*=  *RT*ln*K_d_*, where *R* is the gas-constant and *T* is the absolute temperature.

c Relative free energy difference between poses A and B, calculated using the LIE model.^33^

Consistent with the expectations, DA193 did not discriminate significantly between Pose A or Pose B in hMD-2. In contrast, the control simulation of lipid A^hMD-2^ revealed the Pose A (corresponding to PDB code 3FXI) as a preferred orientation in hMD-2, while pose B was disfavored by 1.6 kcal/mol, which is shown by the free energy differences between the two orientations of lipid A in the binding site of hMD-2 (Table 2).

## Discussion

The βGlcN(1↔1)αGlcN-based tetraacylated lipid A mimetics were designed to fit into the binding pocket of MD-2 and to compete with LPS for the same binding site on the co-receptor protein, thereby inhibiting LPS-induced receptor dimerization and the ensuing pro-inflammatory signaling. The molecular shape of the βGlcN(1↔1)αGlcN backbone of conformationally confined lipid A mimetics is supposed to imitate the spatial arrangement of the native βGlcN(1→6)GlcN backbone of hMD-2-bound antagonists Eritoran and lipid IVa disclosed in the co-crystal structures.^20,21^ By fixing the 3D molecular shape of the carbohydrate backbone of tetraacylated lipid A mimetics in an ‘antagonistic’ conformation through application of β,α(1↔1)-linked diglucosamine scaffold, a species-independent (human and mouse) antagonistic activity has been attained.^9^ We proposed that the exposure of the 2 *N*-acyl chain of the agonists lipid A^hMD-2^/lipid IVa^mMD-2^ on the surface of MD-2, which is responsible for the formation of the homodimeric MD-2·TLR4 lipid A/(lipid IVa) complex and initiation of the innate immune signaling, is enabled by a ‘flipped’ orientation of the proximal GlcN moiety of the β(1→6) diglucosamine backbone of MD-2-bound lipid A as observed in the co-crystal structures of the agonistic ligands (Figure 2A). Such ‘flipped’ or ‘twisted’ orientation is assisted by the inherent flexibility of the three-bond β(1→6) glycosidic linkage of the native diglucosamine backbone of lipid A. As a consequence, upon binding by MD-2, the agonistic lipid A adopts an orientation wherein the ‘flipped’ proximal (reducing) GlcN ring bearing 2 *N*-acyl residue is situated over the shallower part of the hydrophobic groove of MD-2 and faces the secondary dimerization interface (MD-2·TLR4/MD-2*·TLR4*), whereas the tetraacylated distal GlcN moiety (to which the core sugars of LPS are attached) is bound by the deepest and the most hydrophobic part of the MD-2 cleft at the site of the primary dimerization interface (MD-2·TLR4). To prevent receptor complex dimerization, the MD-2·TLR4 antagonist should be bound by MD-2 in an opposite way (rotation by 180°). Indeed, the positioning of the β(1→6) diglucosamine-based antagonistic ligands (lipid IVa and Eritoran) in the binding pocket of MD-2 was shown to be inverted by 180° (Figure 2B).^20,21^

Since the βGlcN(1↔1)αGlcN scaffold, wherein the GlcN rings are nearly co-planar oriented, possesses a non-flexible β,α-(1↔1) glycosidic bond, the ‘flipping’of one of the GlcN moieties is not feasible. Therefore, the antagonistic nature of βGlcN(1↔1)αGlcN LAMs was supposed to be not dependent on the orientation of their carbohydrate backbone in the binding pocket of MD-2 (Figure 2C). As validated by molecular dynamics simulation, all four β-hydroxyacyl chains of DA193 in both binding orientations were fully inserted into the hydrophobic pocket of hMD-2 and the whole molecule was shifted much deeper into the binding cleft compared to the agonist *E. coli* lipid A (Figure 1A). Ionic interactions are known to contribute significantly to the recognition and binding of lipid A variants by MD-2·TLR4 receptor complex and the absence of one of the phosphates strongly impedes the expression of both agonistic and antagonistic activities.^8,46^ Indeed, DA193 formed significantly higher numbers of ionic bridges at the rim of the binding groove of MD-2 than the native ligands (lipid A and lipid IVa), which potentially relates to its stronger interaction with the protein (Table 1; Figure 9). Inward rearrangement of Phe126 residue of MD-2 upon binding of the agonistic ligands was shown to stabilize the exposure of a single acyl chain of lipid A on the surface of MD-2, thereby triggering the formation of an active [MD-2·TLR4–LPS]_2_ complex,^18,47^ whereas outward orientation of Phe126 should prevent homodimerization. Along these lines, the outward positioning of Phe126 in the simulated DA193^hMD-2^ structures in both binding orientations is in full agreement with the experimentally confirmed antagonistic action of DA193 (Table 1).

A 3D molecular shape of the β,α(1↔1)-linked diglucosamine backbone of lipid A mimetics has been ascertained by NOESY experiments of three variably acylated compounds. A *syn*Φα/*syn*Φβ geometry about nonreducing glycosidic linkage was corroborated by the high intensity of the H^1^α/H^1^β cross peaks in the NOESY NMR spectra for three variably acylated βGlcN(1↔1)αGlcN disaccharides (Figures 7 and 8). Thus, a double exo-anomeric conformation around both glycosidic torsions, which was not dependent on the length of the substituting lipid chains, has been established. The experimentally obtained conformation of the βGlcN(1↔1)αGlcN scaffold was confirmed by molecular dynamics simulation (Figure 8).

We have demonstrated that novel synthetic βGlcN(1↔1)αGlcN LAMs potently inhibit LPS-induced pro-inflammatory signaling in human DCs, the human macrophage-like THP-1 cell line and human epithelial cells. The anti-endotoxic potency of the βGlcN(1↔1)αGlcN LAMs declined, in general, with the shortening of the length of (*R*)-3-hydroxyacyl chains, emphasizing the importance of hydrophobic interactions for the ligand binding by hMD-2. The effect of the hydrophobic volume of the glycolipids on the affinity to hMD-2 was the most prominent at lower concentration (100 ng/ml) of antagonists applied for the inhibition of LPS-induced signaling in THP-1 macrophages (TNF-α) and DCs (TNF-α, IL-6, IL-10 and IL-12) (Figures 3 and 5). At increased concentrations (500 and 1000 ng/ml) the differences in the antagonistic effects were not as pronounced, such that nearly total inhibition was provided by all compounds independently of the lipid chain length [except for the short-chain DA257 (4 × C_10_), which was largely inactive at all concentrations tested]. Interestingly, the inhibition of IL-8 production in epithelial cells by variably acylated antagonists was marginally dependent on the chain length, providing excellent results (at least 80% inhibition) for four compounds out of six at concentrations of 100 and 1000 ng/ml, again with exclusion of DA257 (Figure 6). These dissimilarities are obviously related to the differences in the expression of other proteins involved in the LPS recognition cascade (such as mCD-14, which is not expressed in epithelial cells). Thus, in the absence of mCD-14, it is probable that the monomeric LPS molecule could not be as efficiently transferred to MD-2, such that the inhibition of the pro-inflammatory signaling can be achieved likewise by longer- and shorter-chain lipid A mimetics at a concentration of 100 ng/ml (Figure 6).

The 2 × C_12_, 2 × C_14_-acylated compound DA193, which inhibited the expression of the most inflammatory cytokines to the background levels at a concentration 100 ng/ml in all three human cell types tested, was highlighted as the most potent hTLR4 antagonist. Importantly, DA193 was able to inhibit equally the release of IL-6, IL-10, IL-12 and TNF-α in DCs both upon pre-incubation of the cell culture with antagonist (1 h) followed by addition of LPS and upon simultaneous treatment with LPS and DA193 (Figure 5). Powerful suppression of the release of TNF-α in the macrophage-like THP-1 cell line by three (DA193, DA256 and DA253) of six synthetic antagonists was also detected after the pretreatment of the cells with LPS (10 min) (Figure 3). Potentially, this indicates the capability of βGlcN(1↔1)αGlcN LAMs to displace competitively LPS from the binding pocket of hMD-2, which has also been verified by molecular dynamics simulations revealing 20-fold stronger affinity of DA193 to hMD-2 compared with *E. coli* lipid A. The presence of a highly flexible (1→6) glycosidic linkage in the backbone of lipid A and lipid IVa may induce a larger entropic penalty upon ligand binding compared with DA193, which is based on the less flexible βα-(1↔1)-linked diglucosamine scaffold. Thus, the conformational rigidity of the βα-(1↔1)diglucosamine backbone of tetraacylated lipid A mimetics ensures strong binding to MD-2 in both possible orientations (rotation by 180°) of the ligand, which is not the case for native lipid A structures.

In conclusion, synthetic tetraacylated lipid A mimetics wherein the native βGlcN(1→6)GlcN lipid A backbone is displaced by the rigid βGlcN(1↔1)αGlcN scaffold and the labile glycosidic phosphate functionality (as in the native lipid A or in the drug candidate Eritoran) is exchanged for a stable secondary phosphate group, could serve as a basis for development of novel MD-2·TLR4 antagonists with improved anti-inflammatory activities for potential therapeutic application.

## Supplementary Material

Supplementary material
