# Supplementary material for: Anti-endotoxic activity and structural basis for human MD-2·TLR4 antagonism of tetraacylated lipid A mimetics based on βGlcN(1↔1)αGlcN scaffold
Source: Innate Immun. 2015 Jul;21(5):490–503. doi: 10.1177/1753425914550426 (PMC4452626; doi:10.1177/1753425914550426)
Supplement: Supplementary material [file Supplementary_Material.pdf]

Anti-endotoxic Activity and Structural basis for human MD-2·TLR4 Antagonism of Tetraacylated Lipid A Mimetics based on βGlcN(1↔1)αGlcN Scaffold

Jose Antonio Garate, Johannes Stöckl, María del Carmen Fernández-Alonso, Daniel Artner, Mira Haegman, Chris Oostenbrink, Jesús Jiménez-Barbero, Rudi Beyaert, Holger Heine, Paul Kosma, and Alla Zamyatina*

Supporting Information

Table of content

Supplementary Figures and Tables 2
Supplementary Figure S1. 2
Supplementary Figure S2. 3
Supplementary Figure S3. 3
Supplementary Figure S4. 4
Supplementary Table S1. 4
Supplementary Figure S5. 5
Supplementary methods 6
Molecular dynamics simulation: model building 6
Literature 6

Supplementary Figures and Tables

Supplementary Figure S1

A

500 MHz ^{1}H-NMR spectra of DA193, DA256, and DA257 in DMSO at 298 K

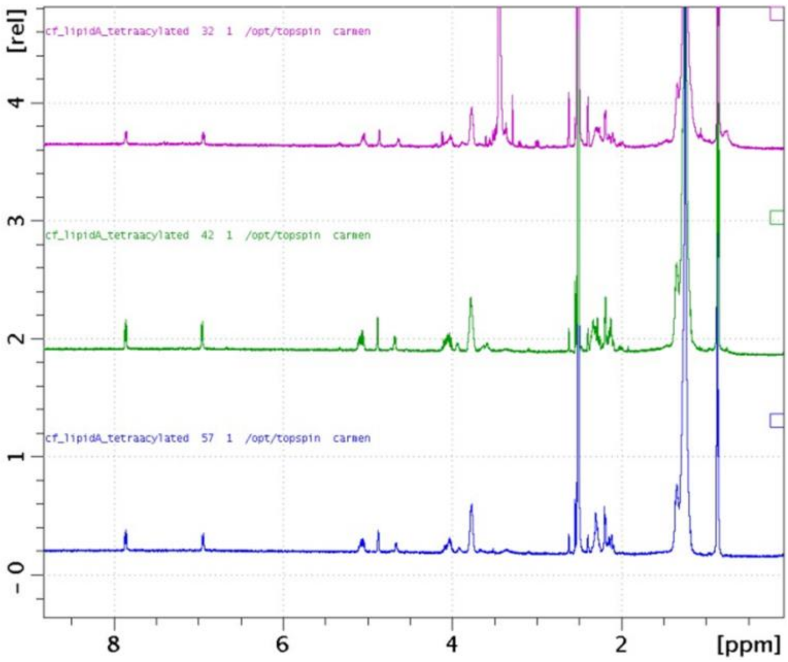

B **^{1}H-NMR spectrum of DA256 (1mM solution) in DMSO-d_6**

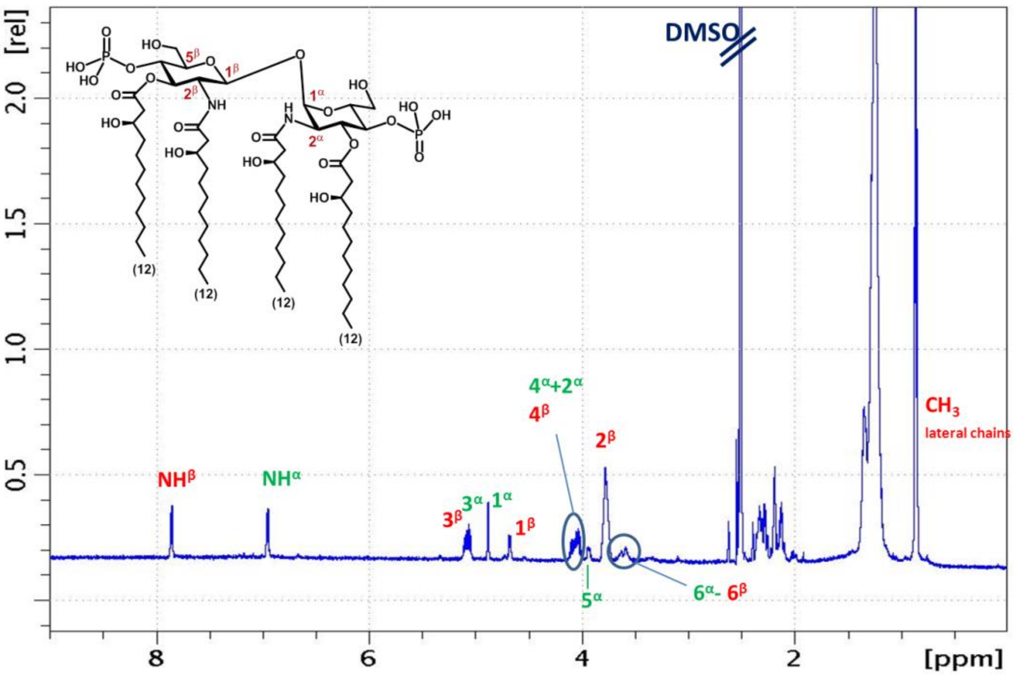

Figure S1. **(A)** Overlay of the 500 MHz ^{1}H NMR spectra of **DA193, DA256 and DA257** in DMSO-d_6 at 298 K. Peak assignments are previously reported;[1] **(B)** ^{1}H-NMR spectrum of **DA256** (1mM solution) in DMSO-d6.

Supplementary Figure S2

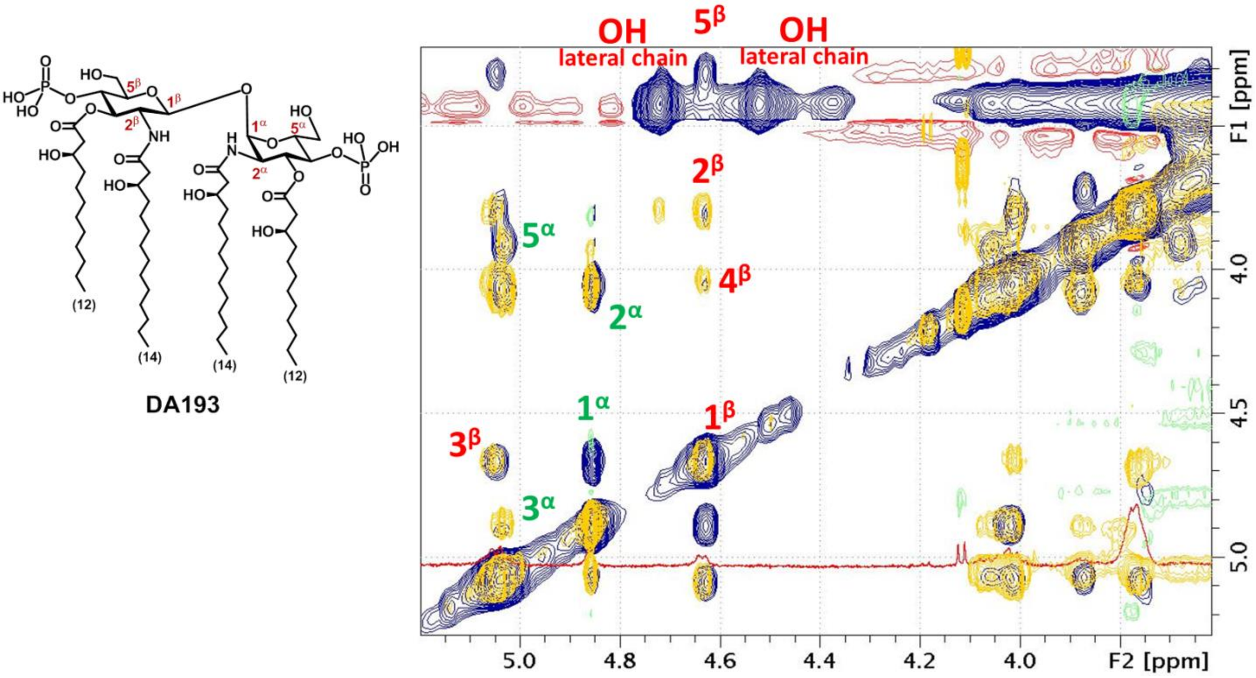

Figure S2. Section of the key region 2D-NOESY spectrum (blue, 300 ms mixing time, 298 K) recorded for **DA193** in DMSO-d6 to delineate the conformation around $\Phi\alpha/\Phi\beta$, superimposed with the corresponding section of the 2D-TOCSY (yellow, 60 ms mixing time, 298 K). Key cross peaks are indicated. A very strong $H^{1\alpha}/H^{1\beta}$ cross peak indicates the existence of one predominant conformation around the $\beta(1\leftrightarrow1)\alpha$ glycosidic linkages.

Supplementary Figure S3.

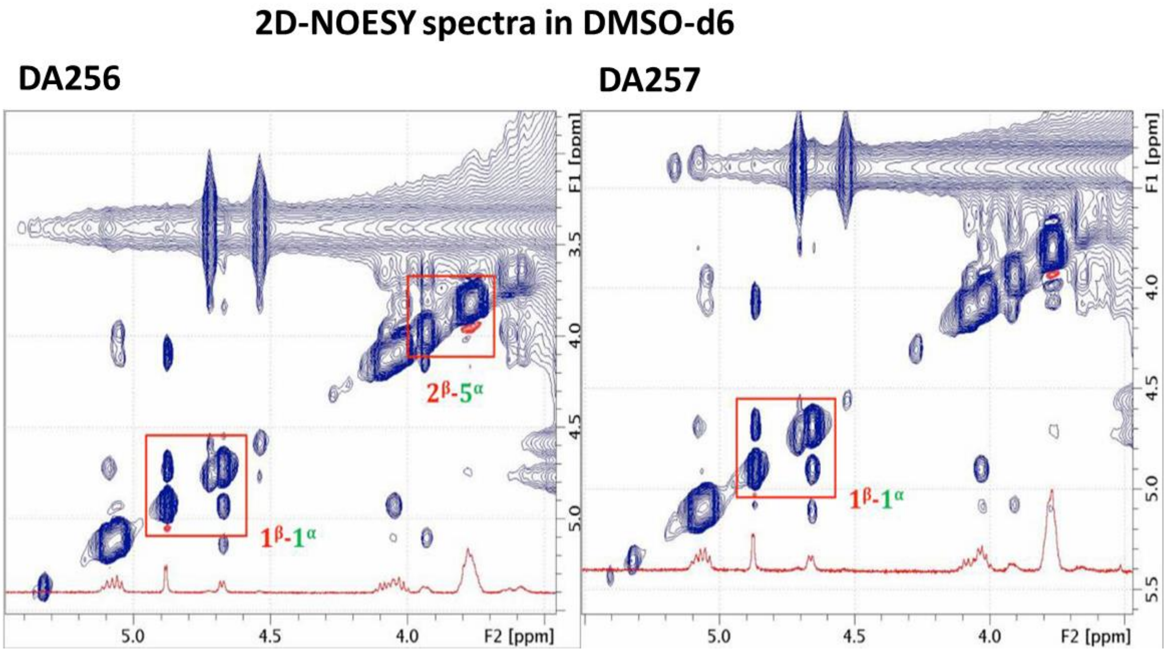

Figure S3. Section of the key region 2D-NOESY spectra (blue, 300 ms mixing time, 298 K) recorded for free **DA256** (left) and **DA257** (right) in DMSO-d_6 to delineate the conformation around syn $\Phi\alpha$/syn$\Phi\beta$. Key cross peaks are indicated. The very strong $H^{1\alpha}/H^{1\beta}$ cross peaks (squared red) in all cases indicate the existence of one predominant conformation around the $\beta(1\leftrightarrow1)\alpha$ glycosidic linkages.

Supplementary Figure S4

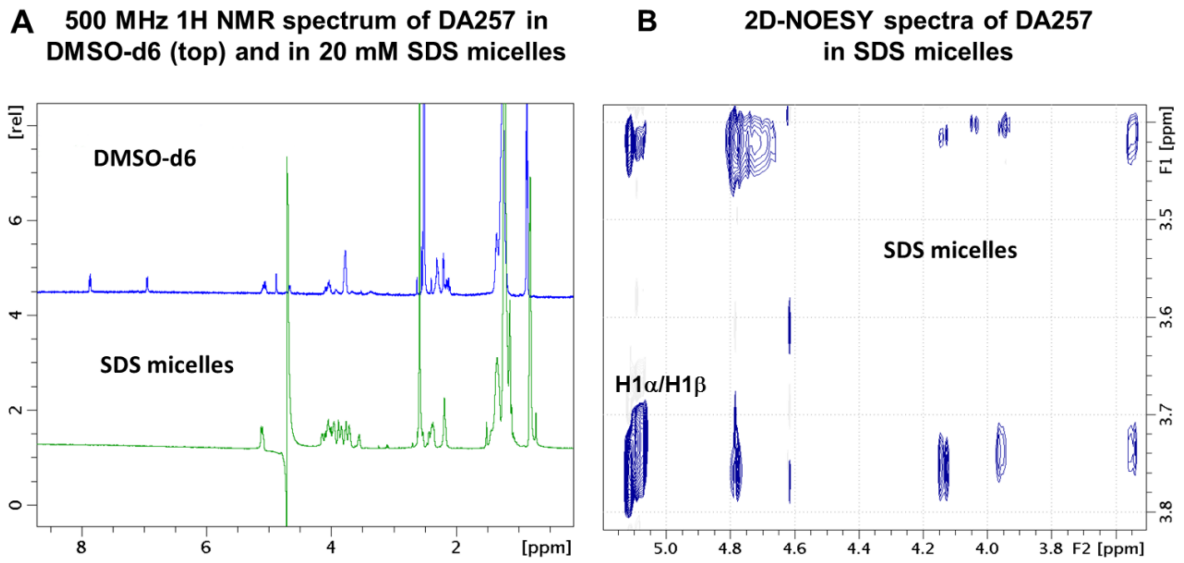

Figure S4: (A) Comparison of the 500 MHz ^{1}H-NMR spectrum (298 K) recorded for **DA257** (0.5 mM) in DMSO-d6 (top) and in 20 mM SDS micelles (bottom). The resolution of the spectrum in the micelle environment is significantly improved; (**B**) Section of the key region 2D-NOESY spectra (250 ms mixing time, 298 K) recorded for **DA257** (0.5 mM) in SDS micelles (20 mM) to delineate the conformation around $\Phi\alpha/\Phi\beta$. The very strong $H^{1\alpha}/H^{1\beta}$ cross peak indicate the existence of one predominant geometry around the $\beta(1\leftrightarrow1)\alpha$ glycosidic linkages.

Supplementary Table S1

SI Table 1: Conformational properties of lipid A, lipid IVa and **DA193** in various molecular dynamics simulations[a]

MD-2 ligand	Enviroment	Orientation (Pose)	P-P distance (nm)	ϕ O_5-C_1-O_1-C_6'(°)	ψ C_1-O_1-C_6'-C_5' (°)	ω O_1-C_6'-C_5'-O_5' (°)
**Lipid A**	water		1.23 ± 0.00	-69± 1	-161 ± 2	-73 ± 1
**Lipid A**	octane/water		1.19 ± 0.01	-67 ± 0	-179 ± 2	-72 ± 0
**Lipid A**	hMD2	**A** as in 3fxi	1.26 ± 0.00	-72 ± 1	-156 ± 1	-72 ± 0
**Lipid A**	hMD2	**B** as in 2E59	1.27 ± 0.00	-73 ± 0	-140 ± 3	-70 ± 0
**Lipid IVa**	water		1.22 ± 0.01	-70 ± 1	-164 ± 5	-73 ± 1
**Lipid IVa**	octane/water		1.20 ± 0.01	-69 ± 1	-172 ± 4	-74 ± 1
**Lipid IVa**	hMD2	**A**	1.21 ± 0.01	-73 ± 1	-168 ± 3	-81 ± 1
**Lipid IVa**	hMD2	**B** as in 2E59	1.21 ± 0.00	-65 ± 1	-168 ± 0	-71 ± 0
				O_5-C_1-O_1-C_1' (°)	C_1-O_1-C_1'-O_5' (°)	
**DA193**	water		1.22 ± 0.00	109 ± 1	-71 ± 0	
**DA193**	octane/water		1.21 ± 0.01	99 ± 2	-65 ± 1	
**DA193**	hMD2	**A**	1.16 ± 0.01	88 ± 3	-58 ± 2	
**DA193**	hMD2	**B**	1.20 ± 0.01	93 ± 3	-65 ± 1	

[a] Error estimates are calculated from block averages

To assess of the accuracy of the simulation of the synthetic antagonist **DA193** in the binding pocket of hMD-2, we have first performed simulations of the native ligands in both orientations (the orientation reported in the co-crystal structures and the opposite) within the binding pocket of hMD-2.

No significant conformational changes in torsional dihedral angles of the glycosidic linkages upon lipid binding by MD-2 compared with simulated unbound state in octane-water interface could be observed (SI Table 1). The average ϕ, ψ, and ω dihedral angles obtained in the simulations of *E. coli* lipid A deviated by 12, 3 and 10°, respectively, from the corresponding angles in the X-ray structure (PDB code: 3FXI). Similarly, the ϕ, ψ, and ω dihedral angles for the simulated lipid IVa^{hMD-2} deviated by 10, 11, and 13 ° from the X-ray conformation (PDB code: 2E59).

Supplementary Figure S5

Since the design of βGlcN(1↔1)αGlcN Lipid A mimetics was based on the crystal structure of β,α-trehalose [βGlc(1↔1)αGlc], we have compared the conformation of the diglucosamine backbone of **DA193** and its glycosidic torsions in a protein-bound and a free state to the conformation of β,α-trehalose disclosed in the crystal structure[3] and confirmed by molecular dynamics simulations.[4] Compound **DA193** was simulated in the binding pocket of hMD-2 considering two different setting of the Φβ/Φα torsions, namely, the torsions restrained to the values found in β,α-trehalose and the torsions released. Our experimental data revealed that the conformation of βGlcN(1↔1)αGlcN scaffold in **DA193^{hMD-2}** having restrained Φα/Φβ torsions was very close to that of β,α–trehalose (SI-Figure 5). As soon as the torsions were released, the spatial arrangement of the GlcN rings in βGlcN(1↔1)αGlcN backbone of **DA193^{hMD-2}** was marginally changed resembling the conformation of the unbound **DA193** at the octane - water interface. Accordingly, the three-dimensional arrangement of the diglucosamine backbone of Lipid A mimetic **DA193** minimized in a free state slightly deviates from that of β,α-trehalose, whereas the former conformation remains preserved in the protein-bound ligand which can be explained by the rigidity of β(1↔1)α glycosidic linkage.

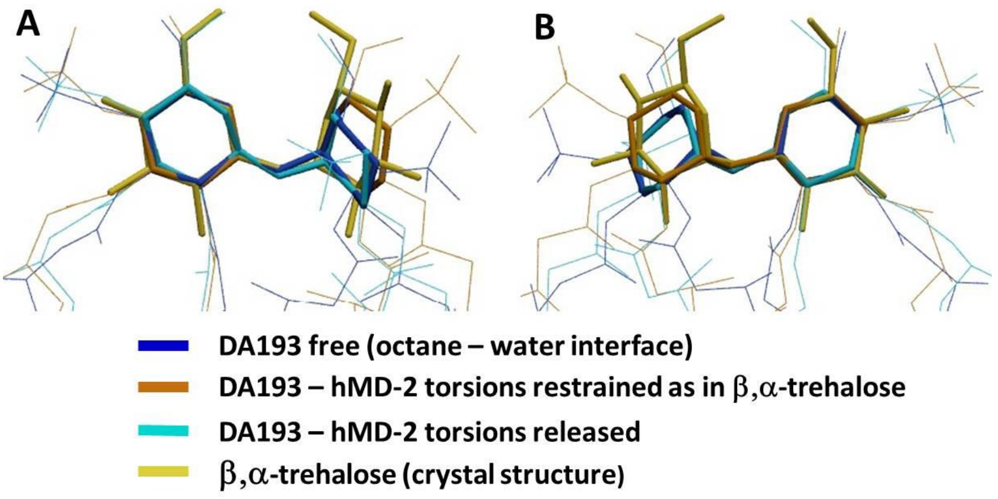

Figure S5. Superimposition of different simulated conformations of the diglucosamine backbone of **DA193** having Φα/Φβ torsions restrained and released with the crystal structure of β,α-trehalose. **(A)** βGlcN /βGlc rings are overlaid, **(B)** αGlcN /αGlc rings are overlaid.

Supplementary methods

Molecular dynamics simulation: model building

Models were based on the crystal structures of hMD2 as available in the Protein databank[5] with PDB entry code 3FXI.[6] Missing hydrogen atoms were added using the internal coordinates of the CHARMM27[8] topology files assuming pH 7.0 for protonation states; the protonation state of all histidines was chosen to be neutral with the proton positioned on N_{δ}. Interactions of lipid A, lipid IVa and **DA193** were described using a combination of the CHARMM36 all-atom carbohydrate force field,[9,10] the CHARMM36 lipid parameter set[11] and TIP3P water model.[12] Force-field parameters for Lipid A, lipid IVa and **DA193** were determined as described earlier.[1,2] The lipids were solvated individually in aqueous surroundings and at a pre-equilibrated octane-water interface. For this periodic cells were constructed with dimensions of 5 x 5 x 5 nm^3, containing approximately 18,000 atoms.

For the complexes of **DA193,** lipid IVa and Lipid A with hMD-2 two orientations of the ligands within the binding cleft of MD-2 were arranged. Position A (***Pose A***) corresponds to the (agonistic) orientation of *E. coli* Lipid A as observed in the co-crystal structures with PDB code 3FXI. For the ***Pose A*** the reducing (proximal) GlcN ring of the diglucosamine backbone of Lipid A (α-GlcN ring) is placed at the top of the shallower part of the binding pocket of hMD-2, thus facing Phe126 loop (the secondary dimerization interface) and the distal β-GlcN ring is placed at the top of the deeper area of the binding pocket of MD-2 (as presented in Figure 2A).[6] For the Lipid A mimetic **DA 193** the ***Pose A*** corresponds to the orientation where the β-GlcN ring is placed at the top of the deepest part of the hydrophobic pocket and α-GlcN ring at the top of the shallower side of the MD-2 pocket, thus facing the Phe126 loop (Figure 2C).

Position B (***Pose B)*** corresponds to the (antagonistic) orientation of lipid VIa (turned by 180° compared to Lipid A^{hMD-2} as in 3FXI) in the co-crystal structure with hMD-2 (PDB code: 2E59) having non-reducing (distal) β–GlcN ring at the top of the shallower part of the pocket and in the close proximity of Phe126 loop of hMD-2 (Figure 2B).[13] For the Lipid A mimetic **DA193** the ***Pose B*** corresponds to the orientation where the β-GlcN ring faces the Phe126 loop (Figure 2C).

For the complexes for which no crystal structure was available, the lipids were manually placed in the active site using the crystal structure with PDB code 3FXI for simulations of hMD-2–bound lipids as templates. The complexes were solvated in periodic simulation boxes of roughly 7.0 x 6.6 x 7.6 nm^3, containing around 32,000 atoms. All systems were electro-neutralized by addition of sodium ions, and built using the program VMDv1.91[14] and the program PackMol.[15]

Literature

1. Artner, D., Oblak, A., Ittig, S., Garate, J.A., Horvat, S., Arrieumerlou, C., Hofinger, A., Oostenbrink, C., Jerala, R., Kosma, P., and Zamyatina, A. Conformationally constrained Lipid A mimetics for exploration of structural basis of TLR4/MD-2 activation by lipopolysaccharide. *ACS Chem.Biol.* 2013; 8: 2423-2432.
2. Garate, J.A., and Oostenbrink, C. Lipid a from lipopolysaccharide recognition: Structure, dynamics and cooperativity by molecular dynamics simulations. *Proteins* 2013; 81: 658-674.
3. Taga, T., Miwa, Y., and Min, Z. α,β-Trehalose monohydrate. *Acta Crystallogr., Sect.C: Cryst.Struct.Commun.* 1997; 53: 234-236.
4. Peric-Hassler, L., Hansen, H.S., Baron, R., and Hünenberger, P.H. Conformational properties of glucose-based disaccharides investigated using molecular dynamics simulations with local elevation umbrella sampling. *Carbohydr.Res.* 2010; 345: 1781-1801.

5. Bernstein, F.C., Koetzle, T.F., Williams, G.J.B., Meyer, J., Brice, M.D., Rodgers, J.R., Kennard, O., Shimanouchi, T., and Tasumi, M. The protein data bank: a computer-based archival file for macromolecular structures. *Arch.Biochem.Biophys.* 1978; 185: 584-591.
6. Park, B.S., Song, D.H., Kim, H.M., Choi, B.S., Lee, H., and Lee, J.O. The structural basis of lipopolysaccharide recognition by the TLR4-MD-2 complex. *Nature* 2009; 458: 1191-1195.
7. Kim, H.M., Park, B.S., Kim, J.-I., Kim, S.E., Lee, J., Oh, S.C., Enkhbayar, P., Matsushima, N., Lee, h., Yoo, O.J., and Lee, J.-O. Crystal Structure of the TLR4-MD-2 Complex with Bound Endotoxin Antagonist Eritoran. *Cell* 2007; 130: 906-917.
8. MacKerell, A.D., Bashford, D., Bellott, Dunbrack, R.L., Evanseck, J.D., Field, M.J., Fischer, S., Gao, J., Guo, H., Ha, S., Joseph-McCarthy, D., Kuchnir, L., Kuczera, K., Lau, F.T.K., Mattos, C., Michnick, S., Ngo, T., Nguyen, D.T., Prodhom, B., Reiher, W.E., Roux, B., Schlenkrich, M., Smith, J.C., Stote, R., Straub, J., Watanabe, M., Wirkiewicz-Kuczera, J., Yin, D., and Karplus, M. All-Atom Empirical Potential for Molecular Modeling and Dynamics Studies of Proteins. *J.Phys.Chem.B* 1998; 102: 3586-3616.
9. Guvench, O., Greene, S.N., Kamath, G., Brady, J.W., Venable, R.M., Pastor, R.W., and MacKerell, A.D. Additive empirical force field for hexopyranose monosaccharides. *J.Comput.Chem.* 2008; 29: 2543-2564.
10. Guvench, O., Hatcher, E., Venable, R.M., Pastor, R.W., and MacKerell, A.D. CHARMM additive all-atom force field for glycosidic linkages between hexopyranoses. *J.Chem.Theory Comput.* 2009; 5: 2353-2370.
11. Klauda, J.B., Venable, R.M., Freites, J.A., O'Connor, J.W., Tobias, D.J., Mondragon-Ramirez, C., Vorobyov, I., MacKerell, A.D., and Pastor, R.W. Update of the CHARMM all-atom additive force field for lipids: validation on six lipid types. *J.Phys.Chem.B* 2010; 114: 7830-7843.
12. Jorgensen, W.L., Chandrasekhar, J., Madura, J.D., Impey, R.W., and Klein, M.L. Comparison of simple potential functions for simulating liquid water. *J.Chem.Phys.* 1983; 79: 926-935.
13. Ohto, U., Fukase, K., Miyake, K., and Satow, Y. Crystal structures of human MD-2 and its complex with antiendotoxic lipid IVa. *Science* 2007; 316: 1632-1634.
14. Humphrey, W., Dalke, A., and Schulten, K. VMD: visual molecular dynamics. *J.Mol.Graph.* 1996; 14: 33-38.
15. Martinez, L., Andrade, R., Birgin, E.G., and Martinez, J.M. PACKMOL: A package for building initial configurations for molecular dynamics simulations. *J.Comput.Chem.* 2009; 30: 2157-2164.
